# Tolerability profile of bevacizumab in metastatic colorectal cancer: about a Medical Department experience

**DOI:** 10.11604/pamj.2016.25.118.2266

**Published:** 2016-10-26

**Authors:** Mohamed Reda Khmamouche, Tarik Mahfoud, Aziz Bazine, Rachid Tanz, Mohamed Ichou, Hassan Errihani

**Affiliations:** 1 Department of Medical Oncology, Military Hospital Mohamed V, Al Irfane, Hay Riad, Rabat, Morocco; 2 Department of Medical oncology, National institute of Oncology, Al Irfane, Hay Riad, Rabat, Morocco

**Keywords:** Colorectal cancer, bevacizumab, tolerability

## Abstract

Colorectal cancer is one of the most common cancers worldwide, and associated with high mortality rates in our country. The prognosis of patients diagnosed with metastatic colorectal cancer (mCRC) has improved markedly over the last 12 years, increasing from 5 months with best supportive care to almost 2 years with combination chemotherapy plus bevacizumab. Bevacizumab is well suited for use in combination with first or second line chemotherapy in the treatment of mCRC because its side effects are predictable and appear not to add to the incidence or severity of the side effects of chemotherapy. The aim of our small study is to explore the tolerability profile of bevacizumab used in daily clinical practice in patients with metastatic colorectal cancer (mCRC) in our department.

## Introduction

Bevacizumab (Avastin*) is a recombinant, humanized anti-vascular endothelial growth factor (VEGF) monoclonal antibody that inhibits tumor angiogenesis. Randomized clinical trials of bevacizumab in combination with oxaliplatin-containing and 5-fluorouracil-based regimens have shown that combination therapy is well tolerated and its toxicity is not substantially greater than that of the chemotherapy alone. They demonstrated significant improvement in survival for patients with mCRC [1–3]. Preliminary data from community-based and observational studies show that the incidence and severity of adverse events with combinations of bevacizumab and newer chemotherapy regimens are similar to those in the pivotal phase III trial with irinotecan, 5-fluorouracil, and leucovorin plus bevacizumab. Across trials, these side effects include a greater risk of grade 3 hypertension and grade 1 or 2 proteinuria, a slight increase in grade 3 or 4 bleeding, and impaired surgical wound healing in patients who undergo surgery during treatment with bevacizumab. [4]. Potentially life-threatening events (arterial thrombotic events, gastrointestinal perforation and fistula formation) have occurred in a small number of patients [5].

## Methods

All patients who received bevacizumab (BV) for mCRC during 4 years in the department of medical oncology at the Military Hospital MOHAMED V in Rabat (Morocco) were analyzed retrospectively. The bevacizumab was administrated with chemotherapy (FOLFOX or XELOX) at 7.5 mg/kg iv over 30-90 min in day 1 every 21 days. For each administration, was assessed the tolerability to bevacizumab perfusion based on patient clinical status, hypertension, proteinuria and other adverse events that were collected in our records.

## Results

Fifty-one patients received bevacizumab for metastatic colorectal cancer during this period. The toxicities observed were minimal proteinuria in 19 cases (37.3%), haemorrhage in 14 cases (27.5%), hypertension in 12 cases (23.5%), venous thrombosis in 4 cases (7, 8%), arterial thrombosis in 2 cases (3.9%), and intestinal perforation in 2 cases (3.9%). However, dyslipidemia was found in 7 cases (13.7%) which represent a new side effect never described in the literature (Table 1).

**Table 1:** Adverse effects observed in our study

Side effects	Percentage
Hypertension grade 1-2	23,5%
Proteinuria grade 1-2	37,3%
Venous thrombosis	7,8%
Arterial thrombosis	3,9%
Hemorrhage	27,5%
Gastro-intestinal perforation	3,9%
**Dyslipidemia**	**13,7%**

## Discussion

The safety profile of bevacizumab in combination with different chemotherapy has been evaluated in clinical trials of Phases II and III in mCRC and during two observational studies, each including about 2000 patients. Some side effects related to bevacizumab were unusual and specific; some of them can be explained by the mechanism of action of this agent: intestinal perforation, delayed wound healing that are related to poor blood supply or bleeding and thromboembolic events [4]. In general, a greater number of grade 3-4 adverse events was observed with bevacizumab + chemotherapy compared to chemotherapy alone. This increase was primarily related to the incidence of hypertension, the most common side effect and most unexpected nearly 40% of patients. The most serious adverse effect on the intestinal perforations that are infrequent (2% of patients), but can be fatal, and arterial thromboembolic events (Table 2).

**Table 2:** Grade 3-4 adverse events observed during trials of bevacizumab in mCRC

Side effects	Bevacizumab + chemotherapy	chemotherapy
Hypertension grade 3-4	22 – 32%	5 -8%
Proteinuria grade 3- 4	23 – 32%	11-22%
Thromboembolic events	18%	16%
Delayed healing of woods	-	-
Surgery under bevacizumab	7-10%	0%
Bleeding, hemorrhage	3- 5%	2-3%
Epistaxis	20 – 40%	0%
Gastrointestinal perforation	1 -2 %	0%

## Conclusion

The use of bevacizumab requires a regular monitoring of blood pressure and quantification of the proteinuria especially in patients who are at greater risk of adverse events. However, monitoring the lipid profile in our patients may be necessary.

### What is known about this topic

- Bevacizumab is anti VEGF used in combination to chemotherapy in the first and second line in the treatment of metastatic colorectal cancer;
- phase III studies demonstrates the benefit of using bevacizumab in this localisation in PFS and OS.

### What this study adds

- The tolerability profile of bevacizumab used in daily clinical practice in patients with metastatic colorectal cancer (mCRC) in our department comparing with literature was comparable.
